# The Bombardment of Coast Towns

**Published:** 1915-02-20

**Authors:** 


### THE BOMBARDMENT OF COAST TOWNS.

### Brighton's Medical Emergency Scheme.

We have received from the Brighton and Hove Medical Emergency Committee the following explanatory memorandum of a scheme for the medical attendance of any persons wounded in consequence of a bombardment by air or by sea of the towns of Brighton and Hove:—

Less than three weeks ago the attention of the Executive Committee of the Brighton Division of the British Medical Association was drawn to the possibility of Brighton and Hove being bombarded from the air and from the sea, and in consequence to the desirability as a precautionary measure that there should be some organised scheme to provide immediate first-aid attendance on the injured. It was decided to approach the Mayors of the two boroughs by deputation and to offer the services of the civilian doctors resident in the two boroughs. It was also pointed out that there would probably be great scarcity of hospital beds, the War Office having so largely called on those available.

The offer made on behalf of the profession to co-operate in any scheme and to give their services gratuitously for first aid was cordially accepted. A conjoint committee was formed of representatives of the local emergency committees of the two boroughs, of the British Red Cross Society, and of the St. John Ambulance, with representatives of the medical profession appointed by the executive committee of the Brighton Division of the Association. As a result the committee has received offers of the assistance of seventy-eight out of ninety civilian doctors, of nurses from the various hospitals and nursing organisations, of stretchers and stretcher-bearers from the Home Protection Brigade, and of over 300 youths from the various boy organisations, who can supply stretchers and act as stretcher-bearers, messengers, and cyclists. The police are co-operating by giving the services of the special constables.

Arrangements have been made with fourteen nursing homes to receive the injured should the supply of beds in the general hospitals fail. The doctors are provided with parcels of emergency dressings and are allowed to use the British Red Cross Society brassard.

The appended scheme has been adopted. Everything is now organised so as to act on the warning signal arranged for. Twenty-nine dressing-stations have been organised. The cost of the scheme will be met through the Borough Councils of Brighton and Hove.

---

### Six Guiding Principles.

The following are the chief principles of the scheme :—

(*a*) That so far as possible all first-aid treatment be given only at a dressing station.

(*b*) That injured persons be invited to go at once to the nearest dressing station, if able to do so.

(*c*) That in the case of those considered unable to do so, a message for a doctor and stretcher be sent at once to the nearest dressing station.

(*d*) That all persons who, in the opinion of the doctor in charge of the station, are able to proceed home after first aid has been rendered, be allowed to do so, and be advised to call in their own doctor later.

(*e*) That those injured persons requiring removal to their own home or to a nursing home or hospital by ambulance be collected at the dressing stations.

(*f*) That the full names and addresses and ultimate destination of all injured persons be registered through the dressing stations.

### The Details (Summarised).

The officer in charge of the Medical Transport will assist with the ambulances at the Central Ambulance Station, so far as his military duties will allow. The hon. secretary of the committee will be in attendance at ——, in order at once to arrange for bed accommodation and to apportion the injured persons amongst them. The supply of ambulances and of beds will be obtainable only through the hon. secretary. Each dressing station will have at its disposal a telephone. Should the telephone service become suspended, cyclists must be used as messengers. All messages given should be in writing. In the event of a hospital, nursing home, or dressing station being rendered unfit to receive the injured owing to destruction by a bomb or otherwise, the staff at the dressing station concerned will, so far as possible, transfer itself to that station which is situated nearest to it, leaving a messenger to advise the public of the change made. Patients should be accumulated at dressing stations to await the ambulance. In all cases the full names and addresses of the injured attended to should be obtained by the doctor in charge of a dressing station or under his direction. Any doctor called to an injured person, whilst he is *en route* to his post, or in consequence of a call sent to his private address, will render first aid if necessary, and later communicate full details to the doctor in charge of the dressing station to which he is attached. Should any one of the public endeavour to requisition an ambulance or stretcher in transit the request must be refused, unless the doctor in charge is satisfied that there is urgency. In case of a death the doctor in attendance should see that the police are advised immediately. All doctors who have volunteered their services will wear the British Red Cross Society brassard.

[A list of the dressing stations is then given.]

### Warning Signal.

If an attack is impending it is most probable that information will be obtained sufficiently in advance to allow of all concerned being warned. This warning will be given during day or night by a signal consisting of three double explosions, with an interval of ten seconds between each pair of explosions.

### Dressing Stations.

The staff and equipment of a dressing station will be: The doctor in charge; one doctor assisting him; one or more nurses; two special constables; a boy clerk; two boy cyclists; four boy messengers; one or more stretchers, with stretcher-bearers; and a supply of the necessary instruments and dressings for first aid.

The doctor in charge must have satisfied himself, by a preliminary visit to his station, that directly the warning signal is given some reliable person appointed by himself will immediately prepare the station. The doctors, nurses, and others appointed, on hearing the warning signal, will immediately proceed to report themselves.

They will not leave subsequently without permission. The staff must be prepared to proceed, on the request of the doctor in charge, to any place indicated in order to render first aid. The detachment sent should consist of a doctor, nurse (if available), stretcher and bearers, and a cyclist messenger. All will return to their dressing station directly their commission has been executed. Should the doctor in charge of the dressing station require further medical assistance after requisitioning one or more of the doctors on his waiting list, he will at once telephone the hon. secretary. The doctor in charge of a dressing station will not close the station until permitted by the officer in charge of the medical transport, and until he is himself satisfied that there is no further call for the services of the staff. He will also make satisfactory arrangements for the station being left as it was prior to occupation, subsequently advising the hon. secretary the cost incurred in so doing. [In the case of ambulance stations analogous rules apply.]

Doctors in attendance at their private addresses, on hearing the warning signal, will immediately return home, and not leave for any private reason before having first of all notified the probable length of absence to the doctor in charge of the dressing station to which they have been attached.

